# Relative Deprivation: How Subjective Experiences of Income Inequality Influence Risk Preferences

**DOI:** 10.3390/bs15040425

**Published:** 2025-03-26

**Authors:** Tae-Young Pak

**Affiliations:** Department of Consumer Science and Convergence Program for Social Innovation, Sungkyunkwan University, Seoul 03063, Republic of Korea; typak@skku.edu

**Keywords:** social comparison, social reference point, reference-dependent utility, relative deprivation, risk aversion

## Abstract

Economic inequality has been linked to changes in individual risk-taking behavior, yet the underlying mechanisms remain underexplored. In this study, I examine whether feelings of relative deprivation from upward social comparisons influence risk preferences. In the randomized experiments, participants were exposed to false information feedback designed to evoke feelings of relative deprivation, and their risk aversion was assessed through hypothetical and incentivized gambles. The results indicate that exposure to relative deprivation reduced risk aversion among men in incentivized lottery experiments, while it had no significant association with risk aversion for either gender in hypothetical gambles. Additionally, relative deprivation lowered perceived social standing and increased anxiety and concerns about personal deservingness—emotional outcomes commonly associated with experiences of relative deprivation. This study provides suggestive evidence that social comparison may influence risk preferences among men through emotional changes and offers insights into how societal inequality affects individual preferences. These findings have important implications for policy interventions aimed at addressing economic disparities and their behavioral consequences.

## 1. Introduction

The behavioral effects of economic inequality have received significant attention in academic research. This issue has become increasingly important as income inequality has continued to rise globally, particularly in the wake of economic disruptions caused by the COVID-19 pandemic and inflationary pressures. Numerous studies have documented that disparities in income distribution are associated with individual decision-making, particularly in contexts involving risk and uncertainty (Brown-Iannuzzi & McKee, 2019; Hopkins, 2018; Mishra et al., 2015; Payne et al., 2017; Pickard et al., 2024; Schmidt et al., 2019). These studies highlight that income inequality is not just a societal issue, but also one with major implications for individual behaviors and preferences. As inequality increases, individuals may perceive greater economic distance from others, and this economic gap becomes more salient and central to decision-making (Sheehy-Skeffington, 2019). In such contexts, individuals may consider riskier choices as an option to close the economic gap and enhance their relative standing within the economic hierarchy (Mishra et al., 2014).

One of the individual-level mechanisms that links economic inequality to risk preferences is the feeling of relative deprivation (RD). Individuals feel relatively deprived when they compare themselves to others within a reference group and perceive themselves as being at a comparative disadvantage (Runciman, 1966). RD tends to be more amplified in conditions of high inequality, as people look up to those at higher levels of the economic hierarchy and feel greater perceived needs (Payne et al., 2017). Social psychologists suggest that this upward social comparison can intensify the motivation to catch up with more advantaged others and increase preferences for riskier options (Smith et al., 2012). Research has shown that feelings of RD are associated with risk-taking behaviors including crime and gambling, where individuals seek high-stakes rewards as a means of compensating for their perceived disadvantage (Brown-Iannuzzi & McKee, 2019; Callan et al., 2008, 2011, 2015; Itskovich, 2024; Keshavarz et al., 2021; Tabri et al., 2015). This behavioral response to RD differs by gender, as men typically exhibit greater risk-taking behavior in competitive and financially uncertain environments (Ermer et al., 2008; Hill & Buss, 2010).

Despite the compelling nature of this mechanism, the issue of how RD affects individual risk preferences remains underexplored. Much of the existing literature has approached this issue through the broader income inequality perspective (Payne et al., 2017; Pickard et al., 2024) or within the framework of reference-dependent utility in game theory contexts (Hopkins, 2018; Linde & Sonnemans, 2012; Müller & Rau, 2019). These studies are limited in that they modeled the association between societal inequality and individual preferences without exploring the underlying individual motives or could not appropriately model upward social comparison within social contexts, which involve a sense of frustration and concerns about personal deservingness. Furthermore, gender differences in responses to RD remain largely unaddressed, although theoretical and empirical evidence suggest that men are more likely to react to RD with riskier choices. This gap in the literature highlights the need for more targeted empirical approaches that directly examine the role of RD and the accompanying psychological effects, particularly in settings where social comparisons and perceived disadvantages are salient.

Therefore, the objective of this study is to examine the effects of RD on risk preferences and the potential gender role. This study employs a standard experimental procedure from social psychology, which is designed to evoke feelings of RD by providing participants with feedback that their reported income is lower than that of comparable others (Callan et al., 2011; Kim et al., 2017; Zhang et al., 2019). By randomly exposing participants to RD priming, this study isolates the causal effects of perceived relative economic disadvantage on individuals’ propensity to take financial risk. Additionally, we examine whether the effects of RD vary by gender, testing whether men exhibit a stronger risk-seeking response than women when confronted with relative economic disadvantage. Our approach allows for a nuanced understanding of how income inequality could alter risk preferences, independent of other confounding factors. Through this analysis, we seek to offer novel insights into the behavioral consequences of economic inequality and inform policy interventions aimed at mitigating its adverse effects.

## 2. Literature Review

### 2.1. Relative Deprivation Theory

In his seminal work, *Relative Deprivation and Social Justice*, Runciman (1966) introduced the concept of RD to describe the feelings of discontent individuals experience when they perceive themselves as unfairly disadvantaged compared to others. He identified four preconditions for RD: (a) lacking a desired resource, (b) awareness that others possess it, (c) desiring the resource, and (d) believing that obtaining it is realistic. Runciman further distinguished between individual (egoistic) RD, where the comparison is individual-level, and group (fraternal) RD, which involves collective comparisons.

The subsequent conceptualization of RD emphasized the interplay of cognitive and affective experiences, where the perception of relative disadvantage incurs cognitive evaluations of fairness and then leads to negative emotions such as anger and resentment (Smith et al., 2012). Social comparison theory suggests that individuals assess their social status by evaluating how they compare to others (Festinger, 1954). Subsequent research has demonstrated that people typically make these comparisons relative to others who share similar characteristics (Gerber et al., 2018). Previous studies linked the experiences of RD to negative psychological outcomes, such as anxiety disorders, depression, and suicidal thoughts (Gero et al., 2017; Lyu & Sun, 2020; Pak & Choung, 2020).

The concept of RD is grounded in a subjective sense of deprivation, which differs from objective disadvantage (Smith et al., 2012). Individuals in objectively deprived situations may not necessarily feel deprived unless a social comparison highlights their comparative disadvantage. For example, individuals with limited financial resources may not recognize their disadvantage until wealthier neighbors move in and make a display of their wealth. Similarly, a blue-collar worker in an affluent area might not view wealthy residents as a relevant comparison group and, thus, may not feel deprived. Runciman (1966) elaborated on this cognitive process by distinguishing two types of reference groups: normative groups, which influence an individual’s norms, values, and attitudes, and comparative groups, which serve as benchmarks for self-evaluation. He emphasized that RD arises from unfavorable comparisons with a comparative group—typically one the individual aspires to catch up with.

RD can be measured with observational data by approximating an individual’s reference group using sociodemographic and geographic characteristics and then assessing economic rank within that group (see Adjaye-Gbewonyo & Kawachi, 2012; D’Ambrosio & Frick, 2012). For instance, researchers might use income data to estimate where an individual stands relative to others in the same region, occupation, or education level, and link this measure to behavioral outcomes to evaluate the effects of RD (Hounkpatin et al., 2020). While this approach provides valuable insights into the contextual and comparative aspects of RD, it has a notable limitation in that it does not capture the subjective elements of RD, such as perceived fairness and concerns about personal deservingness, and the estimated reference group may not fully represent the “correct” reference group to which an individual belongs (Eibner & Evans, 2005; Smith & Pettigrew, 2015). Additionally, unobserved confounders could bias the estimated effects of RD and mislead inferences. In this study, we use an experimental procedure that can precisely manipulate reference group conditions and incur the feelings of RD while confirming its psychological consequences.

### 2.2. Relative Deprivation and Risk Preferences

Expected utility theory assumes consistency in risk preferences, meaning individuals have a stable risk profile that guides their decisions across all situations (see Schildberg-Hörisch, 2018). This view assumes that external factors, such as inequality and social rank, exert minimal influence on preferences, as individual decisions are grounded in a fixed utility function. However, emerging evidence challenges this assumption, indicating that preferences could be systematically affected by social and environmental factors (Callen et al., 2014; Guiso, 2012; Hanaoka et al., 2018; Malmendier & Nagel, 2011).

There is substantial evidence that individual utility is influenced not only by absolute levels of consumption, but also by comparisons to reference points or comparison groups (Kőszegi & Rabin, 2006; Kuhn et al., 2011). The reference point can be socially determined by comparing to others or privately derived from comparisons to one’s past status or expectations (e.g., Loomes & Sugden, 1986; Kőszegi & Rabin, 2006, 2007, 2009). Falling below reference points often results in a heightened sensitivity to losses, which can lead to shifts in risk preferences (Linde & Sonnemans, 2012). For instance, risk sensitivity theory (Mishra et al., 2014, 2015; Mishra & Novakowski, 2016) and prospect theory (Kahneman & Tversky, 1979) suggest that individuals are more likely to adopt riskier strategies when they perceive themselves as falling behind, in an attempt to restore their relative standing. This behavior supports both theoretical accounts (Thaler & Johnson, 1990) and empirical evidence (Odean, 1998) related to reference-dependent utility.

RD theory extends the existing framework by incorporating the broader psychological and emotional consequences of perceived disadvantage. Unlike reference-dependent utility, which typically focuses on fixed reference points, RD emphasizes the subjective and dynamic nature of individuals’ perceptions of circumstances. Recognizing a relatively worse economic position can trigger emotional responses influenced by internalized standards or societal cues. Kahneman’s (2011) dual-system theory highlights how emotionally charged, intuitive reactions (System 1) may overshadow rational, deliberative processes (System 2) in such contexts. This perspective suggests that observed changes in risk preferences under RD reflect context-dependent adaptations rather than shifts in established traits.

Empirical studies show a significant link between personal relative deprivation and risk preferences and behaviors. Mishra et al. (2014) and Mishra and Novakowski (2016) found that competitive disadvantage, a key aspect of RD, increases risk-taking as individuals seek to bridge gaps between their current and desired states, as predicted by risk-sensitivity theory. Linde and Sonnemans (2012) revealed that individuals exhibit different risk behaviors depending on whether they perceive themselves as worse off (loss domain) or better off (gain domain) relative to peers. Lindskog et al. (2022) further highlighted that both the rank and distance from social reference points influence risk-taking. Schmidt et al. (2019) and Schwerter (2024) found that awareness of income inequality and social reference points significantly increase risk-taking, with higher reference points reducing risk aversion. Pak (2023) extended these findings to financial decision-making, showing that RD is negatively associated with risk aversion, but does not consistently translate to risky investment behaviors. Experimental evidence from Dohmen et al. (2021) indicated that workplace risk preferences are shaped by reference points tied to expected earnings, while Fehr and Reichlin (2023) emphasized the role of individual differences, such as locus of control, in moderating RD’s effects.

Gender gradients add an important dimension to understanding RD’s impact on risk preferences. Research in evolutionary psychology suggests that men are generally more sensitive to competitive social rankings and tend to increase risk-taking under conditions of perceived disadvantage (Ermer et al., 2008; Hill & Buss, 2010). Conversely, women may consider relational or communal goals to be as important as financial gains (Chang, 2011). Cultural and societal norms add an additional layer of complexity, as traditional gender role could emphasize a particular gender’s responsibility in financial stuffs (e.g., males in Asian culture), which could alter each gender’s response to RD (Stansbury et al., 2023; Byrnes et al., 1999). Understanding these gender dynamics provides important context for interpreting individual differences in risk preferences.

Considering prior research on RD and risk preferences, we propose the following hypotheses.

**H1.**  *RD reduces risk aversion*.

**H2.**  *The effect of RD on risk aversion is more pronounced for men than women*.

The experimental procedure to evaluate these hypotheses is presented in the next section.

## 3. Methods

### 3.1. Study Design and Data Collection

Two separate studies were conducted to evaluate the research questions. Study 1 used scenario-based, hypothetical gambles over lifetime income to elicit risk aversion (Barsky et al., 1997). Study 2 used the multiple price listing design by Holt and Laury (2002), which offers actual payoffs based on lottery choice experiments. In each study, participants were randomly assigned to one of the two experimental groups (RD group vs. non-RD group) for a 2 × 2 between-subject design.

Participants were recruited from a pool of market research panel members in South Korea. The sample was restricted to working-age adults (25 to 59 years old) with a regular income source, as the treatment priming is based on income differences between participants. To determine the sample size, we aimed to recruit at least 100 participants per gender in each experimental group (1 − β > 0.8; r = 0.30 for a medium-sized effect). In each study, we sampled an additional 100 participants to account for potential dropouts due to irregular responses to the risk aversion questions. Therefore, each study recruited 300 participants for each group and 600 participants in total.

The email link was randomly distributed to Macromill Embrain panel members who met the inclusion criteria (aged 25–59 with regular income). The recruitment process continued until the allocated quota was reached, where the quota was determined by the age, gender, and provincial distribution of the target population. The survey was conducted online in August 2024. All participants were paid between $1 and $2 for completing the survey. Our analyses used a final sample that removes missing values and irregular responses (Table A1 in Appendix A). The study design was IRB-approved (no. 2024-07-081).

### 3.2. Experimental Procedures

Participants were informed that they would be answering questions about financial capability and attitudes. Initially, they were asked to provide their demographic and socioeconomic information, and then manually enter their income, spending, and discretionary income in an open-ended format. After that, participants were shown a message stating that the system would match them with other similar participants based on their input values and calculate a “comparative discretionary income index,” a measure of one’s standing in terms of discretionary income (Callan et al., 2011; Kim et al., 2017; Zhang et al., 2019). The next screen displayed an animated progress bar with a percentage figure increasing from 0 to 100, which helps participants to believe that the system is indeed processing some information. Upon clicking the “next” button, participants were presented with a message indicating that their discretionary income was either 1036 thousand KRW (about 752 USD) less or 172 thousand KRW (about 125 USD) more than comparable others. Those who received a negative index constitute the RD group or treatment group, where the information could be interpreted as a lower economic standing relative to similar others. Participants who received a slightly positive figure form the non-RD group or control group.

After receiving this message, as a manipulation check, participants were asked to rate their perceived economic standing, concerns about personal deservingness, and feelings of anxiety. To measure concerns about deservingness and anxiety, we utilized the five-item scale for concerns about personal deservingness (Callan et al., 2008; Zhang et al., 2019) and the 20-item Spielberger State Anxiety Inventory (Forgays et al., 1998).^1^ Participants were debriefed at the conclusion of the study to ensure that they understood the nature and purpose of the false information feedback and the overall study design.

### 3.3. Risk Aversion

In study 1, participants were presented with a series of hypothetical scenarios, which involve the two jobs with different risk (Barsky et al., 1997).

*“Suppose that you are the only income earner in the family, and you have a good job.... You are given the opportunity to take a new and equally good job, with a 50–50 chance that it will double your income and a 50–50 chance that it will reduce your income by a third. Would you take the new job?”*

Individuals who initially chose the first job were asked to consider a job with a smaller downside risk of one-fourth, while those who selected the second job were presented with a riskier option that could reduce their income by one-half. If participants declined the first job with a one-fourth downside risk, they were subsequently offered a job with only a one-tenth downside risk. Similarly, those who accepted the job with a potential 50% income reduction were further asked about an even riskier job with a downside risk of three-quarters. These five questions resulted in six possible categories of risk aversion, providing a coarse ranking of the individual’s willingness to avoid financial risk (see Appendix B). These rankings and the downside risks for each condition establish the lower and upper bounds on relative risk aversion (Table 1).

In study 2, participants played a series of lottery choice games for real monetary rewards. In each game, they chose between option A and option B, with each option representing a lottery that pays out one of two possible amounts. Option A is designed to be a safer bet with less variability in payoffs (1000 or 800 KRW). Option B is the riskier choice with greater upside and downside risk in payoffs (1900 or 50 KRW). Participants received compensation according to the outcomes of their selected lotteries. The payments were provided in coupon format by the survey administrator.

The original elicitation method involved 10 choices with varying probabilities of winning the lottery (Holt & Laury, 2002). To avoid potential bias due to survey fatigue, this study employed a six-category design with winning probabilities of q = 0.3, 0.4, 0.5, 0.6, 0.7, and 0.8, excluding the top and bottom lotteries where participants are unlikely to switch their choices (Table 2). The order of choices is randomized to remove anchoring and order effects in response (Bosch-Domènech & Silvestre, 2013).

The multiple price list design has advantages over the hypothetical income gamble in that it offers more salient choice sets and can yield risk-loving cases. By making sequential lottery choices, participants reveal the point at which they switch from the safe bet to the risky lottery, which can be converted to a range of risk aversion parameters. Table 3 shows the bounds for risk aversion parameters based on these lottery choices. Appendix B presents a more detailed discussion of this elicitation method.

### 3.4. Interval Regression

This study used interval regression to evaluate the effects of experimentally induced RD on risk aversion. Interval regression is particularly useful when the exact values of the outcome variable are unknown, but confined within known bounds (Long, 1997). This method extends traditional linear regression by incorporating interval-censored data, which allow for more accurate parameter estimation under conditions of partial observation. The model parameters were estimated using maximum likelihood estimation (MLE) to ensure that the predicted values adhere to the specified intervals. MLE is preferred in this context for its robustness and efficiency in parameter estimation, even when data are interval-censored.

This study also included an ordinal outcome (perceived social status) and continuous outcomes (personal deservingness and anxiety), which were used as manipulation checks. These outcomes were modeled using ordered logistic regression and linear regression, respectively. The analyses were performed using the *intreg*, *ologit*, and *reg* commands in Stata SE 17.0 (StataCorp, College Station, TX, USA).

## 4. Results

Table 4 presents the results from the main regressions and the corresponding manipulation checks. All regressions controlled for individual covariates, but these estimates were omitted for brevity. The Cronbach’s alpha is 0.78 for personal deservingness and 0.93 for anxiety. These measures had average variance extracted of 0.52 and 0.79. The background of these scales and measurement items are presented in Appendix B (Table A2 and Table A3).

For risk aversion (column 1), RD does not have a statistically significant effect in study 1 (β = −0.342, *p* > 0.10), suggesting that RD does not strongly influence risk preference in hypothetical scenarios. However, in study 2, where risk preference was measured using an incentivized lottery with real monetary stakes, RD significantly reduced risk aversion (β = −0.210, *p* < 0.01). This indicates that participants exposed to RD are more likely to take financial risks when faced with real, tangible outcomes.

For perceived social status (column 2), RD consistently and significantly lowers participants’ self-reported social standing in both studies, with coefficient estimates of β = −0.594 (*p* < 0.01) in study 1 and β = −0.453 (*p* < 0.01) in study 2. These results confirm that the experimental manipulation of RD effectively created a sense of diminished social rank, validating the effectiveness of the feedback provided during the experiment.

RD also significantly increases concerns about personal deservingness (column 3), with coefficient estimates of β = 0.434 (*p* < 0.01) in study 1 and β = 0.359 (*p* < 0.01) in study 2. These findings suggest that RD triggers a stronger sense of inequity or unfairness, further validating the emotional salience of the manipulation. Similarly, RD significantly elevates levels of anxiety (column 4), with coefficient estimates of β = 0.219 (*p* < 0.01) in study 1 and β = 0.152 (*p* < 0.01) in study 2. These psychological impacts of RD provide robust evidence that the manipulation not only influenced participants’ perceived social standing, but also elicited strong emotional responses.

Table 5 further explores gender differences in the impact of RD on risk aversion. In study 1, RD does not have a statistically significant effect on risk aversion for either men (β = −0.563, *p* > 0.10) or women (β = −0.250, *p* > 0.10), indicating that hypothetical scenarios do not elicit strong behavioral differences. However, in study 2, RD significantly reduces risk aversion for men (β = −0.307, *p* < 0.01), while the effect for women remains negative, but is statistically insignificant (β = −0.094, *p* > 0.10). These results suggest a gendered response to RD in incentivized settings, with men demonstrating a stronger propensity to take financial risks when exposed to RD.

## 5. Discussion

This study proposed that feelings of RD may serve as a mechanism linking the growing inequality to greater tolerance for financial risks. To test this hypothesis, we provided participants with information feedback intended to induce feelings of RD and observed its impact on risk aversion. The results indicate that while RD does not affect risk aversion in hypothetical scenarios, it does lead to lower risk aversion when real financial rewards are involved, particularly among male participants. This study also confirmed that RD effectively lowers perceived social status and heightens concerns about personal deservingness and anxiety, as predicted by risk sensitivity theory and reference-dependent utility.

Our findings complement previous research on social comparison and risk preferences. Schwerter (2024) highlighted that individuals are more likely to take risks if they have the potential to surpass a peer in social ranking. In Schmidt et al. (2019), high-wage individuals were more inclined to take risks when they were aware of wage disparities. Similarly, Mishra and Novakowski (2016) demonstrated that individuals exposed to inequality are more likely to engage in risk-taking to bridge the gap between themselves and wealthier peers. Studies like Fehr and Reichlin (2023) and Lindskog et al. (2022) further explored how social reference points alter risk preferences, with individuals taking more risks to avoid lagging behind others. Notably, Pak (2023) found a significant association between income difference relative to a reference group and self-reported risk aversion. This study adds to the literature by providing experimental evidence on shifting risk preferences in response to RD and the potential gender role in this association.

The null finding in study 1 could be attributed to the use of hypothetical scenarios (without real monetary rewards) to measure risk aversion. Exposure to RD may have heightened sensitivity to actual monetary outcomes, making participants respond in a more risk-seeking manner over lotteries. In contrast, hypothetical scenarios may not have triggered the same psychological responses because the consequences are perceived as less impactful and relevant. Another possibility is that the results in study 2 were driven by risk-neutral or moderately risk-loving participants to take more risk in response to the RD priming. The hypothetical income gamble design does not yield any risk-loving cases, so this likely increase in risk-taking among risk-loving individuals is not captured in study 1.

South Korea has relatively low female labor force participation compared to men (Stansbury et al., 2023), and many women may not feel the same pressure to achieve economic success. This economic environment could encourage men to be more competitive and achievement-oriented, especially in financial and professional domains (Byrnes et al., 1999). RD then might have a stronger psychological impact on men, as it heightens concerns about social ranking. Women, by contrast, may be more influenced by other aspects of social identity, such as social relationships or personal life (Chang, 2011). Our findings on gender gradients corroborate previous research showing greater risk-taking behavior among men in social contexts (Ermer et al., 2008; Hill & Buss, 2010; Pak, 2023; Schmidt et al., 2021).

This study’s findings contribute to the literature by offering novel insights into the psychological mechanisms that link societal economic inequality to risk preferences. Unlike many prior studies that rely on observational data, this research directly tests the causal impact of RD using online experiments. Conceptually, this study expands our understanding of how social comparisons shape individual decision-making, particularly in contexts involving financial risk. Our findings challenge the assumption of stable risk preferences and support theories of reference-dependent utility and risk sensitivity by demonstrating psychological responses to social comparison. From a managerial perspective, financial institutions could use these insights to design personalized investment strategies and risk management tools that account for how social comparisons influence financial behavior. Policymakers may also consider financial education programs and behavioral nudges that reduce inequality-driven risk-taking, particularly among vulnerable groups.

However, the study is not without its limitations. First, the comparison group was composed of anonymous survey participants, which could be perceived as less relevant and salient compared to close others within a social group. Second, this study operationalized RD through the relative shortfall of income, which could overlook other important dimensions beyond income, such as wealth, leisure, and social relationships. Third, the risk aversion measures used in this study could capture risk perception or risk attitudes, not purely stable preferences. While the incentivized design helps mitigate this concern, future research could complement these measures with behavioral tasks or real-world financial decision data to further validate the findings. Lastly, the findings are based on a South Korean sample, which may reflect cultural or economic factors unique to this context. Future research could build on this work by examining these additional dimensions and exploring the extent to which the findings apply across different cultural and socioeconomic settings.

## Figures and Tables

**Table 1:** Risk aversion measured by hypothetical income gamble.

Downside Risk of Risky Job			
Accepted	Rejected	Proportion of Subjects	Range of Relative Risk Aversion
3/4	none	0.14	*r* < 0.31
1/2	3/4	0.11	0.31 < *r* < 1.00
1/3	1/2	0.15	1.00 < *r* < 2.00
1/5	1/3	0.14	2.00 < *r* < 3.76
1/10	1/5	0.15	3.76 < *r* < 7.53
none	1/10	0.31	*r* > 7.53

**Table 2:** Lottery choice experiment.

Decision	Option A	Option B	E(A)–E(B)
1	$2.00 if the die is 1–3 $1.60 if the die is 4–10	$3.85 if the die is 1–3 $0.10 if the die is 4–10	$0.50
2	$2.00 if the die is 1–4 $1.60 if the die is 5–10	$3.85 if the die is 1–4 $0.10 if the die is 5–10	$0.16
3	$2.00 if the die is 1–5 $1.60 if the die is 6–10	$3.85 if the die is 1–5 $0.10 if the die is 6–10	−$0.18
4	$2.00 if the die is 1–6 $1.60 if the die is 7–10	$3.85 if the die is 1–6 $0.10 if the die is 7–10	−$0.51
5	$2.00 if the die is 1–7 $1.60 if the die is 8–10	$3.85 if the die is 1–7 $0.10 if the die is 8–10	−$0.85
6	$2.00 if the die is 1–8 $1.60 if the die is 9–10	$3.85 if the die is 1–8 $0.10 if the die is 9–10	−$1.18

**Table 3:** Risk aversion measured by the multiple price listing design.

No. of Safe Choices	Proportion of Subjects	Range of Relative Risk Aversion
0	0.11	*r* < −0.49
1	0.05	−0.49 < *r* < −0.15
2	0.10	−0.15 < *r* < 0.15
3	0.09	0.15 < *r* < 0.41
4	0.15	0.41 < *r* < 0.68
5	0.13	0.68 < *r* < 0.97
6	0.37	0.97 < *r*

**Table 4:** Main regression results and manipulation checks.

	(1)	(2)	(3)	(4)
Outcome:	Risk Aversion	Perceived Social Status	Personal Deservingness	Anxiety
Study 1:				
RD	−0.342	−0.594 ***	0.434 ***	0.219 ***
	(0.436)	(0.168)	(0.072)	(0.058)
Study 2:				
RD	−0.210 ***	−0.453 ***	0.359 ***	0.152 ***
	(0.079)	(0.169)	(0.072)	(0.052)

Notes: Column (1) reports estimates from interval regression; column (2) reports estimates from ordered logistic regression; and columns (3) and (4) present estimates from linear regression. Standard errors in parentheses. Regressions control for age, gender, education, marital status, employment, income, assets, and home ownership. *** *p* < 0.01.

**Table 5:** Effects of RD on risk aversion by gender.

	(1)	(2)	(3)	(4)
Outcome:	Risk Aversion	Risk Aversion	Risk Aversion	Risk Aversion
	Men (N = 286)	Women (N = 276)	Men (N = 298)	Women (N = 286)
Study 1:				
RD	−0.563	−0.250		
	(0.564)	(0.682)		
Study 2:				
RD			−0.307 ***	−0.094
			(0.111)	(0.112)

Notes: Interval regressions are estimated separately for each gender. Standard errors in parentheses. Regressions control for age, gender, education, marital status, employment, income, assets, and home ownership. *** *p* < 0.01.

## Data Availability

The original contributions presented in this study are included in the article. Further inquiries can be directed to the corresponding author.

## Note

1 See Table A2 and Table A3 in Appendix B for more information on these scales.

## Appendix A. Data Description

**Table A1:** Sample characteristics.

	Study 1 (N = 564)		Study 2 (N = 584)	
	Mean or Freq.	%	Mean or Freq.	%
Age (25–59)	42.7		43.3	
Gender				
Male	288	51.1	298	51.0
Female	276	48.9	286	49.0
Marital status				
Married	318	56.4	335	57.4
Not married	246	43.6	249	42.6
Education				
High school graduate	164	29.1	171	29.3
College graduate	332	58.9	348	59.6
Post-graduate degree	68	12.1	65	11.1
Employment				
Self-employed	46	8.2	63	10.8
Full-time worker	494	87.6	476	81.5
Part-time worker	24	4.3	45	7.7
Monthly income	427.6		422.6	
Own home	325	57.6	334	57.2
Wealth				
<100 mil. KRW	142	25.2	165	28.3
100–300 mil. KRW	173	30.7	170	29.1
300–500 mil. KRW	105	18.6	96	16.4
>500 mil. KRW	144	25.5	153	26.2

## Appendix B. Measures and Measurement Items

### Appendix B.1. Personal Deservingness

The personal deservingness scale is a psychological measure designed to assess individuals’ perceptions of their own deservingness in relation to others (Callan et al., 2008; Zhang et al., 2019). This scale is typically employed to evaluate how strongly individuals believe they deserve rewards, resources, or positive outcomes compared to others. It consists of the five measurement items that are designed to assess feelings of entitlement or beliefs about fairness in social contexts. These items are as follows.

**Table A2:** Items to measure personal deservingness.

Item	Scoring
When you think about the discretionary income you received compared to similar individuals, how satisfied are you?	7-point Likert scale (reverse scored)
When you think about the discretionary income you received compared to similar individuals, how resentful are you?	7-point Likert scale
When you think about the discretionary income you received compared to similar individuals, to what degree do you feel that you want more discretionary income?	7-point Likert scale
When you think about the discretionary income you received compared to similar individuals, to what degree do you feel you deserved your discretionary income?	7-point Likert scale (reverse scored)
When you think about the discretionary income you received compared to similar individuals, to what extent do you think that it is just that you receive this level of discretionary income?	7-point Likert scale (reverse scored)

### Appendix B.2. 20-Item Spielberger State Anxiety Inventory

The 20-item Spielberger State Anxiety Inventory is a widely used psychological instrument designed to measure the current level of anxiety that an individual is experiencing at a particular moment (Forgays et al., 1998). It is a part of the larger State-Trait Anxiety Inventory (STAI), which also includes a trait anxiety scale. The state component of the STAI assesses temporary feelings of anxiety that fluctuate based on the situation or circumstances. It captures how anxious a person feels “right now,” at the time of responding. The state anxiety scale consists of 20 items, which describe a specific emotion or symptom related to anxiety. The scale includes the following measurement items.

**Table A3:** Items in Spielberger State Anxiety Inventory.

Item	Scoring
I feel calm	5-point Likert scale (reverse scored)
I feel secure	5-point Likert scale (reverse scored)
I am tense	5-point Likert scale
I feel strained	5-point Likert scale
I feel at ease	5-point Likert scale (reverse scored)
I feel upset	5-point Likert scale
I am presently worrying over possible misfortunes	5-point Likert scale
I feel satisfied	5-point Likert scale (reverse scored)
I feel frightened	5-point Likert scale
I feel comfortable	5-point Likert scale (reverse scored)
I feel self-confident	5-point Likert scale (reverse scored)
I feel nervous	5-point Likert scale
I am jittery	5-point Likert scale
I feel indecisive	5-point Likert scale
I am relaxed	5-point Likert scale (reverse scored)
I feel content	5-point Likert scale (reverse scored)
I am worried	5-point Likert scale
I feel confused	5-point Likert scale
I feel steady	5-point Likert scale (reverse scored)
I feel pleasant	5-point Likert scale (reverse scored)

### Appendix B.3. Hypothetical Income Gamble Questions to Elicit Risk Aversion

Our income gamble questions to elicit risk aversion are based on a five-question, status-quo-bias free format of risk assessment in the Health and Retirement Study. The status-quo-bias free format is designed to mitigate the influence of respondents’ preference for the current state of affairs, known as status quo bias (Kimball et al., 2008). In the previous version, respondents often favored an option that maintains their current situation and, thus, received a more conservative estimate of their risk aversion.

The hypothetical scenarios frame questions about taking a new job in such a way that respondents are assumed to be changing jobs due to personal reasons, regardless of their satisfaction with a current job. This approach encourages respondents to focus on the risks and benefits of the new job options presented to them, rather than comparing them to their current job, which might be perceived as a safer or more comfortable choice. By removing the direct comparison with their current job, this format aims to provide a more accurate measurement of respondents’ risk aversion by assessing their willingness to accept varying levels of risk in hypothetical job scenarios. The wording of the first question was as follows.
*“1. Suppose you are the only income earner in the family. You recently encountered some personal circumstances that require you to move, and you have to choose between two possible jobs.*
*The first would guarantee your current total family income for life. The second is possibly better paying, but the income is also less certain. There is a 50–50 chance the second job would double your total lifetime income and a 50–50 chance that it would cut it by a third. Which job would you take—the first job or the second job?”*
If respondents answered, “second job,” the interviewer continued with the following.
*“2a. Suppose the chances were 50–50 that the second job would double your lifetime income, and 50–50 that it would cut it in half. Would you take the first job or the second job?”*
If respondents chose “first job” in question 1, they were given an option with a lower downside risk.
*“2b. Suppose the chances were 50–50 that the second job would double your lifetime income and 50–50 that it would cut it by twenty percent. Would you take the first job or the second job?”*
Respondents taking “second job” to question 2a were asked to consider one with a higher downside risk.
*“3a. Suppose the chances were 50–50 that the second job would double your lifetime income and 50–50 that it would cut it by seventy-five percent. Would you take the first job or the second job?”*
Respondents who answered “first job” to question 2b were directed to the following safer alternative.
*“3b. Suppose the chances were 50–50 that the second job would double your lifetime income and 50–50 that it would cut it by 10 percent. Would you take the first job or the second job?”*

Based on their responses to the five questions, respondents are classified into six distinct risk preference categories. These categories are ordered by their level of risk tolerance (or risk aversion) without requiring the assumption of a specific functional form for the utility function. Table 1 in the manuscript provides a summary of these categories and their implications for risk preference.

### Appendix B.4. Multiple Price Listing Lottery

The multiple price listing lottery presents subjects with a series of paired lottery choices, where each pair consists of two different options: a safer option (usually with a lower, but more certain payoff) and a riskier option (with higher potential payoffs, but more uncertainty). In the original Holt and Laury (2002) design, participants make a series of binary choices across 10 lottery pairs with progressively increasing probabilities (Table A4). This change in probability gradually increases the attractiveness of the riskier option, leading to a point where subjects eventually switch from choosing the safe option to the risky one. The switching point indicates the level of relative risk aversion. A participant who switches early (preferring the riskier option sooner) is considered more risk-loving, while a participant who switches later is considered more risk-averse.

**Table A4:** Ten paired lotteries in the multiple price listing design.

Decision	Option A	Option B	E(A)–E(B)
1	1/10 of $2.00, 9/10 of $1.60	1/10 of $3.85, 9/10 of $0.10	$1.17
2	2/10 of $2.00, 8/10 of $1.60	2/10 of $3.85, 8/10 of $0.10	$0.83
3	3/10 of $2.00, 7/10 of $1.60	3/10 of $3.85, 7/10 of $0.10	$0.50
4	4/10 of $2.00, 6/10 of $1.60	4/10 of $3.85, 6/10 of $0.10	$0.16
5	5/10 of $2.00, 5/10 of $1.60	5/10 of $3.85, 5/10 of $0.10	−$0.18
6	6/10 of $2.00, 4/10 of $1.60	6/10 of $3.85, 4/10 of $0.10	−$0.51
7	7/10 of $2.00, 3/10 of $1.60	7/10 of $3.85, 3/10 of $0.10	−$0.85
8	8/10 of $2.00, 2/10 of $1.60	8/10 of $3.85, 2/10 of $0.10	−$1.18
9	9/10 of $2.00, 1/10 of $1.60	9/10 of $3.85, 1/10 of $0.10	−$1.52
10	10/10 of $2.00, 0/10 of $1.60	10/10 of $3.85, 0/10 of $0.10	−$1.85

Note: Reconstructed Table 1 in Holt and Laury (2002).

The 10-lottery design has a potential issue where participants’ responses may be influenced by the context or framing of the choices—a problem known as “embedding bias” (Bosch-Domènech & Silvestre, 2013). This bias can occur because the structure or sequence of the lottery choices might inadvertently lead participants to adopt a different response pattern, affecting the assessment of their risk aversion. For example, knowing that the probabilities in the lotteries gradually increase, participants may anchor their decisions on the initial safe option and gradually become more inclined to take risks as the sequence progresses. Additionally, participants might feel a social or experimental expectation to switch at a certain point due to the increasing probabilities, even if their true risk preference would suggest otherwise. Subsequent research suggests shuffling the order of lotteries and reducing the number of lottery pairs to five or six to reduce decision fatigue (Amador-Hidalgo et al., 2021; Estepa-Mohedano & Espinosa, 2023; Herranz-Zarzoso et al., 2020). This study also randomized the order and used only six lotteries after removing those where decisions were unlikely to switch (i.e., the top and bottom lotteries). This lottery design and the expected payoffs are presented in Table 2 in the manuscript.

### Appendix B.5. Mapping Survey Responses to Risk Preferences

Expected utility theory provides a conceptual basis to convert survey responses to gamble questions into a standardized measure of risk preference, known as the coefficient of relative risk aversion. In this study, we assumed the utility of constant relative risk aversion (CRRA), $U(w)=\frac{w^{1-r}}{1-r}$, where w is the payoff in the lottery and r is the coefficient of relative risk aversion. This utility function captures the curvature of preferences over risky outcomes, with higher values of r indicating greater risk aversion. For a lottery with two outcomes, the expected utility is $EU\left(lottery\right)=p\times U\left(w_{1}\right)+(1-p)\times U\left(w_{2}\right)$, where *p* is the probability of outcome $w_{1}$ and (1−p) is the probability of outcome $w_{2}$. This equation is set equal to the utility of certain payoff and solved for r that makes respondents indifferent between the gamble and the certain amount.

To demonstrate this mapping, consider a respondent who falls into category 4 in the hypothetical income gamble experiment. In this case, the lower bound of the CRRA is determined by solving for r that makes respondents indifferent between the two jobs in question 1:

$$0.5\left(\frac{2^{\left(1-r\right)}}{1-r}\right)+0.5\left(\frac{{\left(2/3\right)}^{\left(1-r\right)}}{1-r}\right)=\left(\frac{1^{\left(1-r\right)}}{1-r}\right)\Rightarrow r\approx 2$$

Likewise, the upper bound of the CRRA is determined by finding r that makes indifferent between two options in question 2b:

$$0.5\left(\frac{2^{\left(1-r\right)}}{1-r}\right)+0.5\left(\frac{{\left(4/5\right)}^{\left(1-r\right)}}{1-r}\right)=\left(\frac{1^{\left(1-r\right)}}{1-r}\right)\Rightarrow r\approx 3.76$$

The risk aversion parameter for these respondents lies in the range of 2 to 3.76. We can get a set of ranges for the CRRA over the seven possible risk categories, as in Table 3.

Applying this mapping procedure to the multiple price listing experiment leads to the similar set of risk aversion ranges. For example, there could be a respondent who chooses option A (safer option) in decision 1 and 2, and then switches to option B (risky option) in subsequent decisions. In this case, the lower bound of the CRRA is:

$$0.4\left(\frac{2^{\left(1-r\right)}}{1-r}\right)+0.6\left(\frac{1.6^{\left(1-r\right)}}{1-r}\right)=0.4\left(\frac{3.85^{\left(1-r\right)}}{1-r}\right)+0.6\left(\frac{0.1^{\left(1-r\right)}}{1-r}\right)\Rightarrow r\approx -0.15$$

and the upper bound of the CRRA is:

$$0.5\left(\frac{2^{\left(1-r\right)}}{1-r}\right)+0.5\left(\frac{1.6^{\left(1-r\right)}}{1-r}\right)=0.5\left(\frac{3.85^{\left(1-r\right)}}{1-r}\right)+0.5\left(\frac{0.1^{\left(1-r\right)}}{1-r}\right)\Rightarrow r\approx 0.15$$

The negative values of r represent risk-loving preferences, positive values indicate risk-averse preferences, and r=0 represents risk-neutral preferences. The inverse of this parameter, 1/r, represents the coefficient of relative risk tolerance (CRRT), which measures an individual’s willingness to take financial risks.
